# Miscellany

**Published:** 1887-08

**Authors:** 


### Miscellany.

Number of Students in German Universities.—The number of students of medicine in the various universities during the past winter *semester* was as follows:

| | | | |
|---|---|---|---|
| Vienna | 2,318 | Fribourg | 428 |
| Munich | 1,350 | Breslau | 362 |
| Berlin | 1,297 | Halle | 315 |
| Wurtzburg | 935 | Bonn | 292 |
| Dorpat | 868 | Marbourg | 271 |
| Leipzig | 781 | Erlangen | 267 |
| Graz | 548 | Zurich | 241 |
| Greiswald | 441 | Kœnigsberg | 237 |
| Tubingen | 235 | Kiel | 234 |
| Gœttingen | 233 | Strasbourg | 233 |
| Innsbruch | 231 | Berne | 227 |
| Jena | 210 | Heidelberg | 202 |
| Giessen | 138 | Basle | 131 |

In the Paris *salon* for 1877 is a picture entitled, "A Clinic at the Salpetriere," representing Charcot giving a clinical lecture on hysteria.

The most interesting feature of the painting is a young girl in the arms of Dr. Babinski, Charcot's assistant. She is having a violent convulsion and has succeeded in disrobing herself almost to the point of nudity, while the throng of students are eagerly watching the symptoms of the disease (?) There is also in the *salon* a picture entitled, "Before the Operation," representing the surgeon Péan lecturing to his class, and a picture of Prof. Peter, the opponent of Pasteur. The French medical press justly condemns this unique method of advertising as derogatory to the dignity of the profession. It is claimed that the picture of the girl affected with hysteria has taught many a mademoiselle to have convulsions in the latest and most approved fashion.

---

"MEDICAL CLASSICS" is the name of a new medical journal, the first number of which has just reached our table. The journal is largely composed of selections from the writings of the fathers of our art. We believe that this is an enterprise which deserves the support of the profession. We devote altogether too much time to trial of new drugs and the study of new theories, while no attention whatever is paid to the history of medicine. In our profession, as in other departments of life-work—

> "We watch the wheels of Nature's plan
> And learn the future by the past of man."

We notice some quotations in the original Greek which will be of especial interest to some of our local physicians who demonstrated, by the recent civil-service examination, that they have no knowledge of the English tongue.

---

PASTEUR'S METHOD.—The English Commission has reported to the home government that Pasteur's method is as truly a preventive of rabies as Jenner's method of vaccination is of small-pox.

Over against this we may present the report of the Austrian Commission made by Prof. Grisch:

"1. The method of Pasteur is still too imperfect to insure to animals an immunity from rabies.

"2. Preventive inoculations practiced upon men who have been bitten by rabid dogs is not based upon well-determined facts.

"3. The preventive method of Pasteur may possibly produce rabies in a previously healthy person."

On the occasion of the ninth convocation of the International Medical Congress, at Washington, a large and capable staff of medically-educated stenographic reporters, under the supervision of a responsible chief, will be in attendance, in the interests of *The Medical Record.* They will prepare a condensed report each day of the proceedings of the general sessions and various sections, which, with graphic accounts of the social incidents, and interesting items of news and gossip, will each day be telegraphed to the publication office of *The Medical Record,* in New York, and immediately prepared by the editorial staff for publication in its current issues. By the kindness of *The Record* the Journal will receive these reports.

---

Tablet Triturates.—Physicians are becoming more and more appreciative of the advantages which these elegant preparations afford. Messrs. Wyeth Brothers' advertisement in this number furnishes a list, which embraces every drug now demanded, and the doses are so graduated as to meet every indication. It is obvious that, in the making of these preparations, the physician is compelled to rely upon the integrity of the manufacturer. We have used Wyeth's triturates largely, and they have never failed to give us the expected effect. The writer has personally visited the laboratory of this house, and the confidence inspired then by the perfection of the processes employed in manufacture, and the quality of the drugs and chemicals inspected, has been sustained in every case by the use of these preparations in actual practice.

---

Treatment of Diarrhœa by Iodoform and Charcoal.—Picchini treated eight cases of diarrhœa, with symptoms of fermentation, with the following:

| | | |
|---|---|---|
| ℞ | Iodoform, . . . . | grs. 9. |
| | Ether, . . . . . | ℥ 3⅛. |
| | Vegetable charcoal, finely powdered, . | ℥ 3⅛. |
| | Glycerin, ad. . . . . | ℥ 12. |

The iodoform must be dissolved in the ether, and the powdered charcoal thoroughly mixed. After the ether has evaporated, the glycerin should be added. To take in twenty-four hours, by teaspoonful or tablespoonful, suspended in a glass of water.—*Journal de Médecine.*

Jules Simon recommends the following in the treatment of infantile eclampsia:

| | |
|---|---|
| Musk, . . . . . | gr. iij. |
| Camphor, . . . . . | gr. xv. |
| Chloral hydrat., . . . . | gr. viiss. |
| Yolk of egg, . . . . . | no. i. |
| Distilled water, . . . . | ℥ iv. |

M. Sig.—Use as enema after the intestines have been emptied.

---

Budin gives the statistics of lying-in department of *La Charite* as follows:

| | |
|---|---|
| Total number of confinements during the four years from 1883 to 1886 inclusive, . . | 1,349 |
| Total deaths, . . | 21 or 1.58 per ct. |
| Deaths from infectious diseases, | 9 or 0.66 per ct. |
| Deaths from infectious diseases contracted in hospital, . | 4 or 0.29 per ct. |

---

Permanganate of potash is the latest remedy for malarial fever. The discovery of its curative properties in these affections was made by Dr. I Vroz, of the Virgin Islands, who observed that women suffering from malarial affections and dysmenorrhœa were cured of both diseases by the administration of from one-half a grain to a grain of potassium permanganate three times a day.

---

Salkowski and Wolf have recently been studying some cases of fatal poisoning from the use of mollucs. Even common oysters and clams may be poisonous if these shell fish are grown in stagnant water or in motionless water of harbors. The poison is contained exclusively in the liver and is destroyed by the action of heat and by soaking in a solution of bicarbonate of soda.

---

Balsherolski has made a comparative study of the antiseptic values of the biniodide and bichloride of mercury. He concludes that the bichloride is more easily managed and is more efficient for the destruction of the bacillus of carbuncle. The biniodide, on the other hand, is more destructive to the bacteria of suppuration in staphylococci.

Pityriasis Versicolor.—Besnier of Paris recommends the following pomade in the treatment of this obstinate affection :

| | |
|---|---|
| Acid salicylic, . . . | gr. xlv. |
| Sulphur precipitat., . . . . . | ʒ iiss. |
| Lanolini. | |
| Vaselini, . . . . . | āā ʒ iss. |

M. Sig.—Apply thoroughly after washing part affected.

---

The Norwegian Government has taken another step towards discovering the origin and nature of leprosy, which is so common on the west coast of Norway, by dispatching Dr. G. A. Hansen, director of the Leprosy Hospital at Bergen, to this country, for the purpose of inquiring into the heredity of the disease among Scandinavian emigrants to the United States.

---

Prof. A. Vogel has observed that plants do not always contain their characteristic alkaloids when grown under other than natural conditions. Hemlock does not yield conine in Scotland, and cinchona plants are nearly free from quinine when grown in hot-houses. Tannin is found in the greatest quantities in trees which have had a full supply of direct sunlight.

---

A writer in an exchange informs us that on several occasions he has prescribed belladonna for sterile women, and has found that, after taking it for several weeks, they have become pregnant. *Moral*—Unmarried women should let belladonna severely alone !—*Ex.*

---

Medical Logic.—A physician observed that 20 per cent. of the men present at a grand opera were bald-headed. A few nights afterward he noticed that but 5 per cent. of the men at a comic opera were bald-headed. *Ergo*, comic opera is a hair restorer.

---

A Russian surgeon, Jarochewski, has discovered that strychnine will counteract the narcotic effects of alcohol. Large quantities of alcohol may be imbibed without producing intoxication, provided strychnine is given at the same time.

---

A Georgia doctor has recently successfully performed a cæsarian section on a cow. Mother and calf are both doing well.

A TOOTH immersed in a solution of tincture ferri chloridi, one to eight, will have its enamel entirely destroyed in one hour. In prescribing tincture of iron, therefore, alcohol, glycerine or syrup should be employed as a vehicle.

---

DR. V. HORSELEY has succeeded in removing a tumor from the dorsal portion of the spinal cord. The diagnosis was made by Dr Gowers, who suggested operative interference. The tumor measured 1¼ by 1½ inches.

---

INJECTION FOR GONORRHŒA :

| | | |
|---|---|---|
| Salol, . . . . . | ʒ iiss. | |
| Gum. acaciæ, . . . . | ʒ iss. | |
| Aq., . . . . . | ℥ iv. | M. |

*World's Med. Review.*

---

THE President has appointed Dr. George M. Sternberg to investigate the subject of inoculation as a preventive in yellow fever, as recently practiced in Brazil and Mexico.

---

AN epizootic of a disease resembling gonorrhœa is prevalent among the cattle of some parts of Switzerland. The disease renders the cows sterile and becomes chronic.

---

LABOULBIM has determined that water is the usual vehicle for the transmission of the eggs of ascarides. The common filter will remove these spores from the water.

---

TWENTY-ONE hospitals of New York city, with the aid of the dispensaries, treated over 122,000 people, at an expense of over $600,000, last year.

---

AN exposition for the study and illustration of infant hygiene is soon to be held in Paris. This is the first enterprise of the sort ever proposed.

---

THE Homœopathic colleges of the United States have graduated 1,996 physicians in the past five years.

WOMEN students are hereafter to be excluded from the German universities.

KINESITHERAPY is the name given by European physicians to the Swedish movement cure.

FRENCH physicians recommend ergotine in the treatment of meningitis and cerebral hemorrhage.

LAC CANINUM (bitch's milk) is a new remedy introduced by a homœopathic physician.

THE University of Nebraska has discontinued its medical department.

SMALL-POX is epidemic in the eastern portion of the island of Cuba.

THERE are twenty-two female dentists in England.

CARL FRIEDLANDER, the pathologist, is dead.

